# Supplementary material for: The Effect of pH and Storage Temperature on the Stability of Emulsions Stabilized by Rapeseed Proteins
Source: Foods. 2021 Jul 18;10(7):1657. doi: 10.3390/foods10071657 (PMC8303418; doi:10.3390/foods10071657)
Supplement: Supplementary file 1 [file foods-10-01657-s001.zip › foods-1292192-supple.pdf]

foods

Supplementary material

The effect of emulsion pH and storage temperature on emulsion stability stabilized by rapeseed protein precipitated at different pH

Karolina Östbring [1*], María Matos[2], Ali Marefati[1], Cecilia Ahlström[1], and Gemma Gutiérrez[2]

[1] Department of Food Technology Engineering and Nutrition, Faculty of Engineering, Lund University, P.O. Box 124, 221 00 Lund, Sweden; ali.marefati@food.lth.se (A.M.); cecilia.ahlstrom@food.lth.se (C.A.)
[2] Department of Chemical and Environmental Engineering, University of Oviedo, Julián Clavería 8, 33006 Oviedo, Spain;
matosmaria@uniovi.es (M.M.); gutierrezgemma@uniovi.es (G.G.)
* Correspondence: karolina.ostbring@food.lth.se

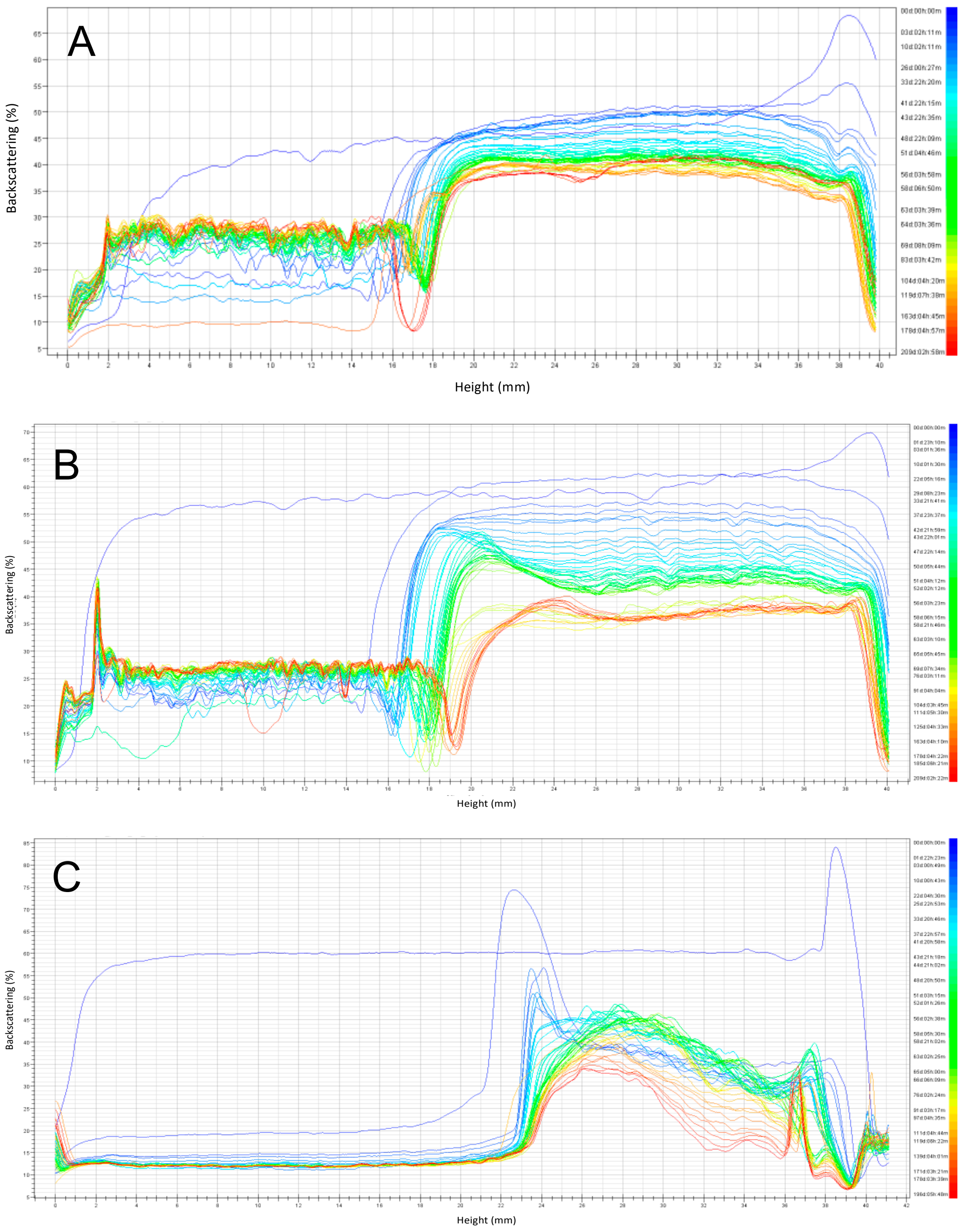

Figure S1. Emulsion backscattering profiles prepared at pH 4.5 and stored for six months at 4°C. (A) Emulsion stabilized by RPP3 (B) Emulsion stabilized by RPP6.5, (C) Emulsion stabilized by soy lecithin.

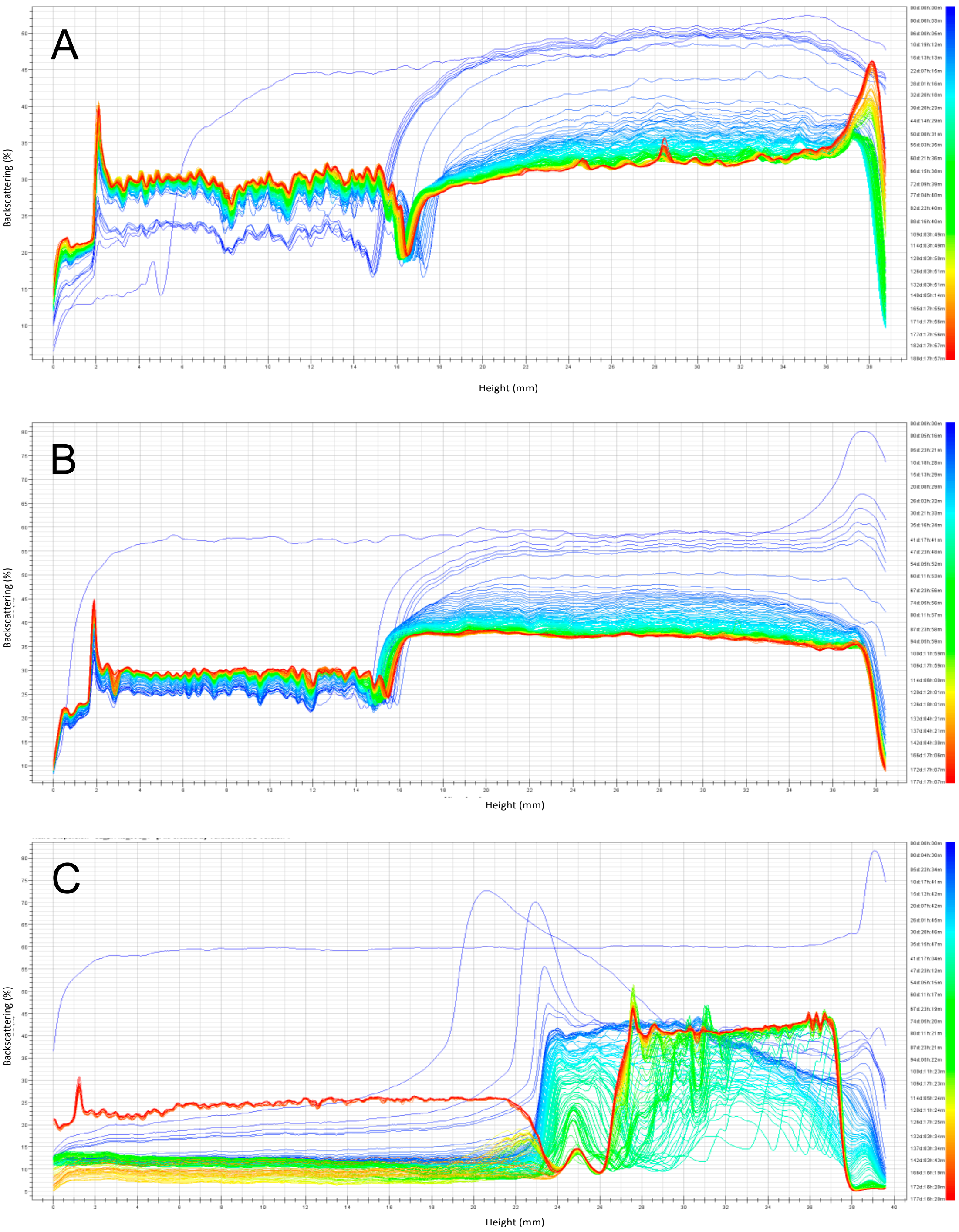

Figure S2. Emulsion backscattering profiles prepared at pH 4.5 and stored for six months at 30°C. (A) Emulsion stabilized by RPP3 (B) Emulsion stabilized by RPP6.5, (C) Emulsion stabilized by soy lecithin.
